# Machine Learning-Based Prediction of Textural Properties and Nonlinear Regulatory Pattern Analysis of 3D-Printed Dough Containing Konjac Glucomannan

**DOI:** 10.3390/foods15111941

**Published:** 2026-06-01

**Authors:** Wenjun Leng, Yilan Sun, Jianhua Xie, Jie Pang

**Affiliations:** 1 College of Food Science, Fujian Agriculture and Forestry University, Fuzhou 350002, China; lwj15003716738@163.com (W.L.); yilansun@126.com (Y.S.); 2 Zhangzhou Food Industrial Technology Research Institute, Zhangzhou 363000, China; 3 College of Food Engineering, Zhangzhou Institute of Technology, Zhangzhou 363000, China; 4 School of Synthetic Biology and Biological Manufacturing, Tianjin University, Tianjin 300072, China

**Keywords:** dough, texture, machine learning, SVR, GPR

## Abstract

The precision of 3D-printed food is dictated by the macroscopic textural stability of the dough system during extrusion. In this study, we investigated the nonlinear regulatory effects of Konjac Glucomannan (KGM) concentration and printing pressure on the textural properties of 3D-printed dough. Using a space-filling experimental design (*n* = 30), Support Vector Regression (SVR) and Gaussian Process Regression (GPR) models were developed to map the complex interactions between formulation and process variables. The results indicated that KGM concentration and printing pressure exhibit significant nonlinear coupling effects on hardness, cohesiveness, and chewiness. After 4-fold cross-validation and systematic hyperparameter optimization, the SVR model demonstrated satisfactory interpolative predictive performance within the investigated parameter space, achieving $R_{p}^{2}$ values of 0.990 for gumminess and 0.987 for chewiness, while the GPR model effectively characterized the predictive uncertainty. Furthermore, the model predicted a favorable processing region (0.5–0.8% KGM and 4.0–4.6 bar) within the investigated design space. This research provides a quantitative, data-driven framework for the formulation pre-optimization of 3D-printed dough under specific experimental settings.

## 1. Introduction

The targeted development of 3D-printed dough products depends not only on microstructural stability but also, and perhaps more critically, on the macroscopic textural characteristics of the post-extrusion system [1]. Effectively elucidating the regulation of the target dough’s textural properties under varying processing conditions can establish a robust foundation for the customized development of 3D-printed foods. Notably, formulation variables (KGM concentration) and mechanical parameters (printing pressure) serve as the primary regulatory indicators determining final product [2] quality. The alterations in shear-thinning behavior induced by KGM concentration interact in a highly nonlinear manner with the extrusion shear forces generated by printing pressure. This leads to complex, multi-index interactions. From a molecular perspective, previous studies have proposed that KGM molecules compete for available water within the gluten-starch matrix, potentially interfering with the formation of the disulfide-bonded gluten network while simultaneously establishing non-covalent cross-links within the polysaccharide framework. Furthermore, during the extrusion process, the dough system is subjected to intense mechanical shear forces generated by the printing pressure [3]. Conventional empirical methods often struggle to capture these deep mapping relationships. Therefore, in this study, advanced machine learning algorithms—specifically Support Vector Regression (SVR) and Gaussian Process Regression (GPR)—were employed. By utilizing KGM concentration and printing pressure as core inputs, and key Texture Profile Analysis (TPA) parameters as target responses, this study aims to decode these nonlinear regulatory patterns.

While contemporary investigations have explored the effects of KGM on the 3D printability of germinated brown rice gels [4] and optimized extrusion printing parameters for dehulled Andean fava bean flours [5], systematic head-to-head comparisons of Support Vector Regression (SVR) and Gaussian Process Regression (GPR) models for textural property prediction in wheat dough systems remain scarce. A comprehensive recent review [6] has further identified predictive uncertainty quantification as a critical unaddressed gap in machine learning-driven 3D food printing research, noting that the vast majority of existing studies generate only deterministic point predictions without quantifying the reliability or confidence bounds of their outputs. Additionally, the predominant experimental design approaches employed in this domain—including conventional response surface methodology and full-factorial designs—exhibit inherent limitations in efficiently capturing complex nonlinear interactions within high-dimensional parameter spaces.

The present study addresses these identified knowledge gaps through a multifaceted approach: (1) elucidating the nonlinear coupling effects between KGM concentration and extrusion printing pressure on dough textural properties; (2) performing a rigorous side-by-side performance comparison of SVR and GPR models for the prediction of key textural attributes; (3) leveraging the probabilistic nature of GPR to quantify predictive uncertainty and generate 95% confidence intervals for all model outputs. This approach provides a robust framework for predicting the mechanical behavior of 3D-printed dough under specific processing constraints, as well as a visual analysis method.

## 2. Theoretical Framework for Machine Learning Predictive Modeling and Research Indices

Within the domain of food science, machine learning has been extensively utilized in applications such as flavor profiling [7], quality grading, and shelf-life prediction. In alignment with the research objectives of this study, two representative nonlinear regression algorithms were selected: Support Vector Regression (SVR) and Gaussian Process Regression (GPR). Their fundamental principles and mathematical formulations are subsequently detailed below.

### 2.1. Data Preprocessing and Normalization

To ensure data integrity, a rigorous quality control protocol was applied to the Texture Profile Analysis (TPA) data. For each formulation, three independent replicates were initially performed. Before calculating the arithmetic mean, each set of replicates was manually screened for technical outliers induced by instrumental fluctuations or sample preparation inconsistencies. This pre-established quality control protocol was uniformly applied across all experimental groups. Specifically, if a single measurement exhibited a coefficient of variation (CV) >10%, that specific trial was invalidated. This 10% CV threshold is a widely accepted industry standard in food materials [8,9], as it effectively filters out technical outliers induced by instrumental fluctuations, sample preparation inconsistencies, or accidental air bubbles entrapped within the dough matrix. In this study, a total of 3 anomalous data points from 3 specific groups were discarded, representing only 3.3% of the total 90 measurements (3 replicates × 30 samples). To maintain statistical independence and data integrity, new independent replicates for these groups were immediately prepared and tested under identical experimental conditions to ensure a reliable triplet of data points. While this quality control procedure improves data reliability, it is important to acknowledge that it may introduce a minor bias toward more consistent measurements. However, this potential bias is negligible in the present study due to the extremely low proportion of discarded data points and the strict adherence to identical experimental protocols during remeasurement. Subsequently, the validated response values were processed via arithmetic mean aggregation to filter out high-frequency physical measurement noise, providing a smoothed baseline for non-linear regression modeling [10].

Furthermore, when machine learning models process multidimensional input features, significant discrepancies often exist in the physical dimensions and numerical ranges across different variables (e.g., KGM concentration ranges from 0 to 1%, while printing pressure ranges from 4.0 to 5.0 bar). To ensure the rigor of the modeling process and prevent data leakage, a stratified data preprocessing strategy was implemented. The MinMaxScaler was first fitted solely on the training dataset to determine the objective feature ranges (*x*_min_ and *x*_max_). Subsequently, these locally derived scaling parameters were employed to transform both the training and the independent test datasets into the dimensionless interval [0, 1]. This approach ensures that the model’s generalization performance is evaluated on truly unseen data. Following the model prediction phase, an inverse transformation was performed using the training-set-based parameters to map the dimensionless outputs back to their original physical units, ensuring the interpretability of the dough textural properties [11,12]:

(1) $$x_{norm}=\frac{x-x_{min}}{x_{max}-x_{min}}$$

where *x* represents the original experimental observation; *x*_min_ and *x*_max_ denote the minimum and maximum values of the variable within the sample set, respectively; and *x*_norm_ signifies the scaled value following normalization. Upon generating predictive outputs, since the values remain within the dimensionless interval of [0, 1], an inverse transformation must be applied to map them back to their original physical dimensions. This step is essential to facilitate subsequent intuitive evaluation and process analysis of the textural characteristics of the 3D-printed dough. The mathematical expression for this inverse transformation is given as follows:

(2) $${\hat{y}}_{real}={\hat{y}}_{norm}\times (y_{max}-y_{min})+y_{min}$$

### 2.2. Space-Filling-Inspired Discrete Experimental Design

In constructing datasets for machine learning predictive models, the sampling strategy fundamentally dictates the model’s computational efficiency and generalization boundaries. The experimental design of the 3D printing parameter space was informed by the space-filling principle characteristic of Latin Hypercube Sampling (LHS) [13]. Instead, a discrete space-filling design was adopted for practical experimental feasibility. Discrete levels of KGM concentration (0%, 0.25%, 0.5%, 0.75%, 1.0%) and printing pressure (4.0, 4.25, 4.5, 4.75, 5.0 bar) were selected to mimic the uniform, non-overlapping distribution typical of LHS, ensuring balanced coverage across the investigated parameter space. This strategic partitioning of the continuous variable space ensures a uniform distribution of samples across the concentration gradients and pressure intervals. The rationale for adopting this space-filling approach is twofold: first, it mitigates the over-concentration of data points in localized regions, thereby empowering the model to capture underlying patterns without predictive blind spots; second, it guarantees that every discrete level of each variable is exhaustively sampled, preventing the information redundancy frequently encountered in traditional full-factorial designs.

(1) Space-filling property: LHS ensures a uniform distribution of samples across the KGM concentration gradient and pressure intervals. This effectively mitigates the over-concentration of data points in localized regions and prevents the emergence of predictive blind spots at the margins, thereby empowering the model to accurately capture and predict underlying patterns.
(2) Prevention of dimensionality collapse: The textural characteristics of 3D-printed dough are synergistically influenced by multiple variables. In scenarios exhibiting disparate parameter sensitivities (e.g., subtle adjustments in printing pressure may exert a less pronounced effect on texture compared to variations in KGM concentration), LHS guarantees that every discrete level of each variable is exhaustively sampled. This circumvents the information redundancy and dimensional feature loss frequently encountered in traditional experimental designs [14].

### 2.3. Support Vector Regression (SVR) Modeling and Nonlinear Textural Mapping

Support Vector Regression (SVR) is a robust algorithm founded upon statistical learning theory and the principle of structural risk minimization. Distinct from conventional regression approaches that aim to minimize the residual sum of squares, the core objective of SVR is to construct an optimal function-fitting hyperplane with a maximized margin. This approach minimizes prediction errors within a predefined tolerance threshold. Furthermore, the introduction of slack variables endows the model with favorable fault tolerance, thereby significantly enhancing its generalization performance.

To endow the model with tolerance against experimental fluctuations, SVR incorporates an ϵ-insensitive loss function, governed by the critical parameter ϵ Its mathematical formulation is rigorously defined as follows:

(3) $$L_{\epsilon }(y,f(x))=\left\{\begin{array}{ll} \;0, & |y-f(x)|\leq \epsilon \\ \;|y-f(x)|-\epsilon , & |y-f(x)|>\epsilon \end{array}\right.$$

**Figure 1:**
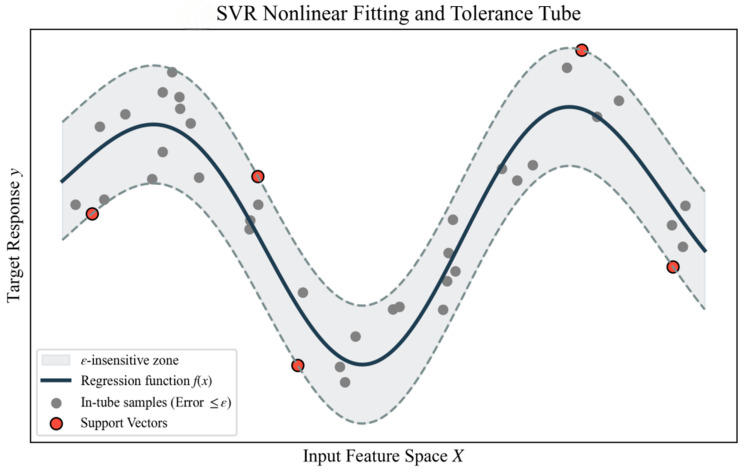
Schematic illustration of SVR nonlinear fitting and the tolerance tube principle. Note: The dashed lines represent the upper and lower boundaries of the ϵ-insensitive tube.

A linear loss is penalized only for the magnitude of the prediction error that falls outside this defined tube boundary. The underlying principle is specifically illustrated in Figure 1.

Building upon the aforementioned loss function definition, and by introducing slack variables *ξ*_i_^*^ and *ξ*_i_ to quantify the actual errors exceeding the upper and lower boundaries of the tube, respectively, the SVR model training is ultimately transformed into solving the following convex quadratic programming problem subject to inequality constraints:

(4) $$\underset{\omega ,b,\xi ,\xi^{*}}{\min}\frac{1}{2}||\omega |{|}^{2}+C\sum_{i=1}^{n}(\xi_{i}+\xi_{i}^{*})$$

(5) $$s.t.\left\{\begin{array}{l} y_{i}-\omega^{T}\phi (x_{i})-b\leq \epsilon +\xi_{i}\\ \omega^{T}\phi (x_{i})+b-y_{i}\leq \epsilon +\xi_{i}^{*}\\ \xi_{i},\xi_{i}^{*}\geq 0,i=1,2,\ldots ,n \end{array}\right.$$

where C represents the penalty parameter, which regulates the trade-off between model complexity and empirical fitting error. This model is capable of effectively capturing the abrupt textural transition trends within the dough [15], which are induced by the reinforcement of physical cross-linking points.

### 2.4. Gaussian Process Regression (GPR) Modeling and Predictive Uncertainty Quantification

**Figure 2:**
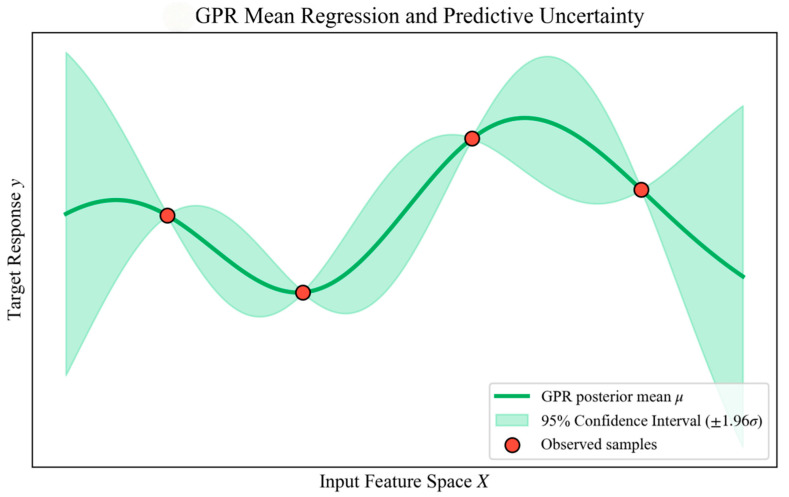
Schematic illustration of GPR mean regression and variance-based uncertainty.

Gaussian Process Regression (GPR) is a non-parametric probabilistic model founded on Bayesian inference. In contrast to SVR, which yields only a single deterministic prediction, the fundamental advantage of GPR lies [16] in its capability to provide a posterior probability distribution for the predicted outcomes alongside performing nonlinear regression [17]. Given that 3D-printed dough constitutes a complex food matrix, its internal water distribution exhibits a multicomponent state (as evidenced by the coexistence of bound and semi-bound water in LF-NMR results), and its microscopic network structure demonstrates distinct pore heterogeneity (as confirmed by SEM observations). This microstructural heterogeneity, dictated by the intrinsic properties of the materials, inevitably engenders inherent fluctuations in the macroscopic textural indices during repeated experimental trials. Consequently, the introduction of a GPR model equipped with uncertainty quantification capabilities can effectively characterize the range of predictive variance induced by these microstructural discrepancies. The conceptual schematic is illustrated in Figure 2.

GPR is a non-parametric Bayesian regression method based on a Gaussian process prior distribution, which outputs both predicted values and corresponding uncertainty intervals. A Gaussian process is defined by its mean function *m(x)* and covariance function (kernel function) (*k*(*x_i_,x_j_*)):

(6) $$\left\{\begin{array}{c} m(x)=E[f(x)]\\ k(x_{i},x_{j})=E\left[\left(f(x_{i})-m(x_{i}))(f(x_{j})-m(x_{j})\right)\right] \end{array}\right.$$

Four typical kernel functions were tested in this study to capture different nonlinear characteristics of dough textural data: squared exponential (SE), Matérn 5/2, rational quadratic (RQ), and RQ-White noise combined kernel.

### 2.5. Hyperparameter Optimization and Model Evaluation Metrics

**Figure 3:**
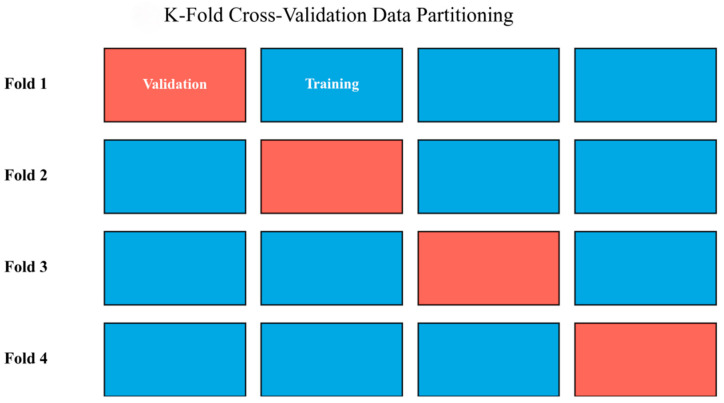
Schematic illustration of the K-fold cross-validation data partitioning mechanism.

The generalization performance of machine learning predictive models is highly contingent upon the configuration of hyperparameters. To prevent model overfitting on the dough textural data within the training set, hyperparameter optimization was performed using a Grid Search combined with K-fold Cross-Test (CV) [13,18]. The training dataset was randomly partitioned into K mutually exclusive subsets (*K* = 4 in this study), with one subset iteratively selected as the test set, as schematically illustrated in Figure 3. For any given parameter combination θ within the search space, the total cross-validation evaluation error, E_cv_(θ), is defined as follows:

(7) $$E_{cv}(\theta )=\frac{1}{K}\sum_{i=1}^{K}E_{i}(\theta )$$

To objectively quantify the predictive accuracy of the SVR and GPR models for various dough textural indicators on an independent test set, the coefficient of determination for calibration ($R_{c}^{2}$) and prediction ($R_{p}^{2}$), root mean square error of cross-validation (RMSECV) and prediction (RMSEP), mean absolute error (MAE), and the ratio of performance to deviation (RPD) were selected as the primary evaluation metrics [15]. RPD is defined as the ratio of the standard deviation (SD) of the reference values in the test set to the RMSEP. Generally, an RPD between 2.0 and 3.0 indicates a good predictive model, while an RPD > 3.0 represents excellent predictive capacity. Their respective mathematical formulations are defined as follows:

(8) $$R^{2}=1-\frac{\sum_{i=1}^{n}(y_{i}-{\hat{y}}_{i}{)}^{2}}{\sum_{i=1}^{n}(y_{i}-\bar{y}{)}^{2}}$$

(9) $$RMSE=\sqrt{\frac{1}{n}\sum_{i=1}^{n}(y_{i}-{\hat{y}}_{i}{)}^{2}}$$

(10) $$MAE=\frac{1}{n}\sum_{i=1}^{n}|y_{i}-{\hat{y}}_{i}|$$

where *n* represents the number of dough samples in the test set; yi denotes the experimental observation of the textural indicator; ${\hat{y}}_{i}$ signifies the predicted value generated by the model; and $\bar{y}$ is the mean of the observed values. Specifically, an R^2^ value approaching 1 indicates a higher explanatory power of the model regarding the textural patterns of dough as regulated by KGM and printing pressure; meanwhile, RMSE and MAE values closer to 0 reflect smaller absolute predictive errors, thereby enhancing the practical significance of the model.

## 3. Materials and Methods

### 3.1. Experimental Materials

High-gluten flour was purchased from Guangdong Xinliang Industrial Co., Ltd. (Shenzhen, China); Konjac glucomannan (KGM) was obtained from RUIBIO (Wuxi) Co., Ltd. (Wuxi, China).

### 3.2. Experimental Instruments

The IMT-MEP-S microfluidic electrostatic 3D printing system (Nanjing Beier Era Technology Co., Ltd., Nanjing, China) and the TMS-PRO texture analyzer (FTC, Sterling, VA, USA) were utilized in this study.

### 3.3. Preparation of Composite Flour and 3D Printing

The datasets for model training and validation were partitioned using a space-filling strategy inspired by Latin Hypercube Sampling (LHS) [19] principle, as detailed in Table 1. This comprised 24 training sets and 6 test sets (specifically, the 3D-0.5% KGM group at 4.5 bar included one standard sampling point and two replicates at the central point).

Initially, precise quantities of KGM were weighed according to Table 1 and incorporated into 50 g of high-gluten flour. The mixtures were homogenized to produce composite powders with varying KGM concentrations (calculated based on the dry weight of the high-gluten flour). Subsequently, 30 mL of deionized water was added to each mixture. The samples were kneaded until the composite powder and gluten proteins were fully hydrated, resulting in a smooth, crack-free dough surface. All treatments were performed in triplicate.

The prepared dough was loaded into the printing syringe and conditioned at 30 °C to ensure optimal dough development. The syringe was then installed in a pneumatic extrusion-based 3D printer equipped with a 2.3 mm inner diameter nozzle and connected to an air compressor. The 3D printing parameters, as specified in Table 1, included a printing speed of 50 mm/min, a 100% infill density, and a layer height of 2.5 mm [20]. The printed specimens were cubes with orthogonal inter-layer filaments, designed using Cartesian coordinates and executed via NcStudio software V5 [21].

### 3.4. Determination of Dough Textural Properties

The textural properties of the 3D-printed dough were characterized using a TMS-PRO texture analyzer. Following the printing process, the specimens were encapsulated in plastic film and equilibrated for 10 min prior to analysis. Texture Profile Analysis (TPA) was performed using a P/36R cylindrical probe to simulate a double-compression cycle. The experimental parameters were set as follows: a crosshead speed of 120 mm/min, a trigger force of 0.05 N, a compression strain of 45%, and a 5 s interval between cycles [22]. Five primary textural parameters were derived: hardness, cohesiveness, springiness, gumminess, and chewiness [23].

### 3.5. Computational Environment and Software

The computational tasks in this study were conducted using the PyCharm 2025.3.1, which served as the primary platform for algorithm development, debugging, and execution. Several industry-standard open-source Python libraries were utilized for scientific computing. The specific development environment is detailed below:

(1) Data Processing and Statistical Analysis: Pandas 2.3.3 and NumPy 2.4.2 libraries were employed for importing raw experimental data, performing matrix transformations, identifying and removing technical outliers based on a coefficient of variation (CV) threshold > 10% prior to calculating the arithmetic mean for parallel replicates, and computing statistical parameters.
(2) Machine Learning Framework: The core algorithmic architecture was developed using the Scikit-learn library (version 1.8.0) in Python (version 3.11). Specifically, hyperparameter optimization was executed using the GridSearchCV function, and the GPR kernels were instantiated via the sklearn.gaussian_process.kernels module. To strictly prevent data leakage and ensure methodological reproducibility, a standardized data preprocessing and modeling pipeline was implemented. Given the distinct physical dimensions and numerical magnitudes of the five TPA indices, independent predictive models were constructed for each textural parameter. The holistic machine learning workflow strictly adhered to the following sequential protocol: initial partitioning of the dataset into training and independent test sets; fitting the MinMaxScaler exclusively on the training set to independently normalize the input features and the specific target variable to a [0, 1] range; conducting hyperparameter optimization via cross-validation and grid search strictly within the scaled training bounds; fitting the final optimal model; and ultimately performing external test set predictions, followed by inverse normalization to restore the target variables to their original physical units. This rigorous pipeline guarantees that no information from the test set influences the model training or scaling processes.

## 4. Results and Discussion

### 4.1. Correlation Between 3D Printing Parameters and Dough Textural Properties

**Figure 4:**
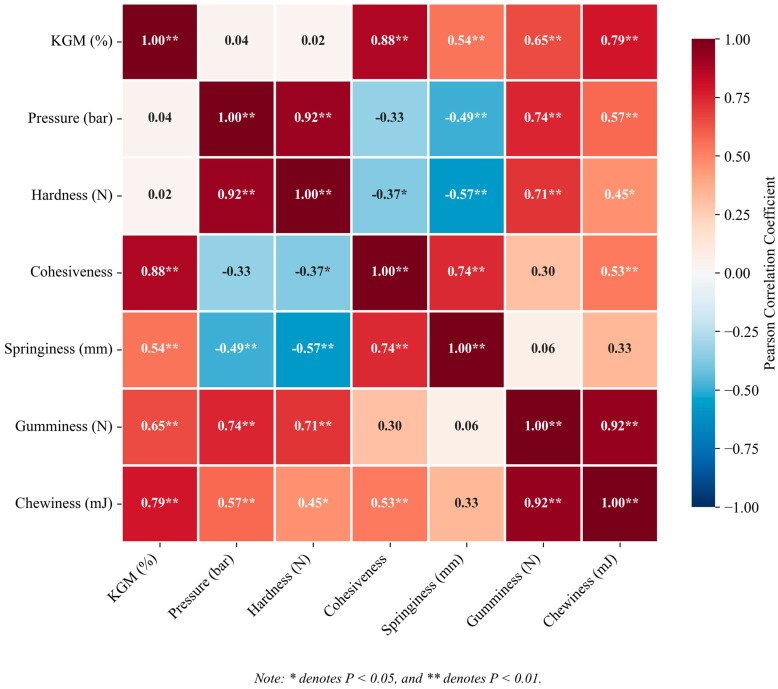
Heatmap of Pearson correlation coefficients between 3D printing parameters and dough textural properties. Note: Color intensity corresponds to correlation magnitude (red = positive, blue = negative).

Understanding the linear associations between process parameters and textural attributes provides a preliminary descriptive overview of the dough system. As illustrated in Figure 4, Pearson correlation analysis revealed that KGM concentration exhibited highly significant positive linear correlations with dough cohesiveness (r = 0.88), chewiness (r = 0.79), gumminess (r = 0.65), and springiness (r = 0.54) (all *p* < 0.01). Mechanistically, this linear trend is hypothesized to be associated with the abundant hydroxyl groups on KGM chains, which potentially facilitate hydrogen-bond cross-linking within the gluten-starch matrix. Simultaneously, its robust water-holding capacity is suggested to enhance the bound-water proportion and restrict moisture mobility [18,19,20,21]. Conversely, printing pressure showed a highly significant positive correlation with hardness (r = 0.92, *p* < 0.01) and a negative correlation with springiness (r = −0.49, *p* < 0.01), possibly reflecting the shear-induced network compaction and suggesting an orientational rearrangement of gluten proteins during extrusion [22]. Additionally, significant linear synergies were observed among the textural parameters themselves, such as between gumminess and chewiness (r = 0.92, *p* < 0.01). This heatmap (*n* = 30) clearly visualizes the strong linear associations between key variables, with all reported correlations reaching at least *p* < 0.05 significance, supporting the reliability of subsequent nonlinear modeling.

However, it must be emphasized that Pearson coefficients exclusively quantify linear dependencies. The macroscopic texture of 3D-printed dough is ultimately the result of a highly dynamic [24], competing, and nonlinear interplay between KGM’s chemical reinforcement and the physical disruption of extrusion shear. Therefore, while this descriptive statistical analysis confirms that formulation and pressure are highly relevant input variables, it fundamentally cannot capture the deep multidimensional mapping relationships. This limitation necessitates the subsequent deployment of advanced non-parametric machine learning algorithms. In this context, SVR and GPR are introduced not only to precisely extract nonlinear features from the limited experimental dataset via kernel functions, but also to construct a robust predictive architecture capable of comprehensively decoding these complex textural regulatory patterns. In addition, it should be noted that these correlation coefficients were calculated based on a relatively small sample size (n = 30). While the observed strong correlations provide valuable insights into the relationships between processing parameters and textural properties, their statistical reliability may be somewhat limited. Therefore, these findings should be interpreted with appropriate caution, and further validation with larger sample sizes is warranted to confirm the robustness of these relationships.

### 4.2. Parameter Optimization Process for SVR and GPR Models

The predictive accuracy and generalization capability of machine learning models are inherently linked to the selection of hyperparameters; inappropriate hyperparameter configurations can result in overfitting or underfitting, thereby failing to capture the nonlinear mapping between 3D printing process parameters and dough textural indices. To eliminate the subjectivity and stochasticity associated with manual tuning and to ensure stable, reproducible model performance, systematic hyperparameter optimization was conducted independently for both the SVR and GPR models. The entire optimization process was confined to the training set; the independent test set remained uninvolved in both model training and hyperparameter selection, serving exclusively for the final validation of generalization performance to prevent inflated performance metrics arising from data leakage.

#### 4.2.1. SVR Hyperparameter Optimization

Drawing upon benchmarks from similar food texture prediction studies and preliminary experimental results, a log-uniform sequence was employed to define the hyperparameter search space [25], ensuring comprehensive coverage across multiple orders of magnitude and avoiding insufficient resolution within intermediate intervals [4]. The optimization ranges were specified as follows: C ∈ [0.1, 1000] (9 logarithmic steps), γ ∈ [0.01, 10] (7 logarithmic steps), and ε ∈ [0.001, 0.1] (5 logarithmic steps).

**Figure 5:**
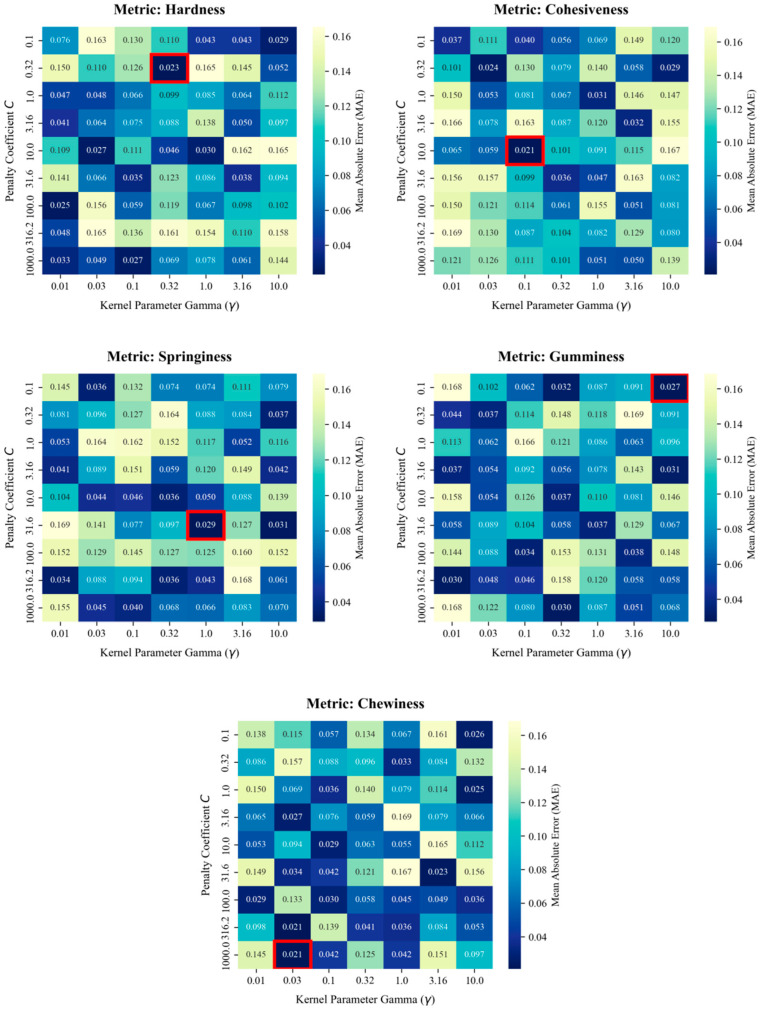
Heatmaps of Mean Absolute Error (MAE) for SVR hyperparameter optimization of 3D-printed dough textural properties. Note: Hyperparameters (C, γ, ε) were optimized via 4-fold cross-validation on the training set (*n* = 24). Darker colors indicate lower MAE (better performance); red boxes mark the optimal hyperparameter combination for each index.

The error heatmaps for SVR hyperparameter optimization are illustrated in Figure 5, where darker colors indicate lower Mean Absolute Error (MAE) and improved fitting performance. The red boxes denote the global optimal hyperparameter combinations for each textural index (MAE values are presented to three decimal places). As observed, the optimal configurations for all textural indices are situated within the central regions of the parameter grids, confirming that the search spaces were appropriately defined to encompass the global optima and avoiding boundary-constrained solutions. Furthermore, the sensitivity to hyperparameters varied significantly among different textural attributes, specifically for hardness, gumminess, and chewiness.

Textural indices exhibited higher sensitivity to variations in the penalty coefficient C, with optimal C values remaining consistently at elevated levels; meanwhile, structural textural parameters, such as cohesiveness and springiness, were more sensitive to the kernel parameter γ, yielding relatively larger optimal γ values. This reflects fundamental differences in the nonlinear characteristics governing the relationships between various textural indices and process parameters.

Table 2 presents the optimal hyperparameters and performance metrics of the SVR model across the five textural indicators. The global optimal hyperparameter configurations identified in Table 2 were employed to construct the final SVR-based textural predictive model.

To rigorously evaluate the predictive robustness of the SVR model, particularly given the characteristic limited sample size (*n* = 30) of 3D printing formulation studies, a 4-fold cross-validation (CV) strategy with randomized data shuffling was implemented. As presented in Table 3, the SVR model exhibited highly stable predictive performance across all five textural parameters. Notably, the mean cross-validation determination coefficients ($R_{CV}^{2}$) for chewiness (0.989), gumminess (0.985), and hardness (0.972) were consistently high. More importantly, the standard deviations (SD) associated with these metrics remained remarkably narrow (all SD ≤ 0.027). This minimal variance confirms that the model’s accuracy is not a byproduct of an arbitrary data split (overfitting), but rather a true reflection of the underlying nonlinear mapping learned by the model. Furthermore, while the absolute values of the Root Mean Square Error (RMSE_CV_) varied among the attributes—for instance, 0.700 for chewiness versus 0.027 for cohesiveness—these variations are strictly proportional to the intrinsic physical scales and units of the respective TPA measurements. Relative to the overall experimental ranges, these prediction errors are marginal, effectively demonstrating that the SVR model is a reliable and robust tool for interpolative texture prediction within the investigated parameter space. The good stability during cross-validation, combined with the high accuracy on the independent testing set ($R_{p}^{2}$ ranging from 0.844 to 0.990; RPD ranging from 2.77 to 10.97), confirms that the optimized architecture effectively captured the underlying nonlinear textural transitions without suffering from overfitting. Notably, gumminess and chewiness demonstrated the highest predictive performance, with $R_{p}^{2}$ reaching 0.990 and 0.987, and RPD values of 10.97 and 9.69, respectively. Although the predictive performance for cohesiveness was relatively lower, it remained well within the accuracy thresholds required for food textural prediction. In conclusion, the model effectively characterizes the nonlinear mapping between KGM concentration, printing pressure, and dough texture, establishing a robust foundation for subsequent model comparison and process optimization.

#### 4.2.2. GPR Hyperparameter Optimization

The kernel function serves as the cornerstone of the Gaussian Process Regression (GPR) model, directly governing its capacity to discern underlying data patterns. Given the characteristic strong nonlinear correlations between process parameters and 3D-printed dough textural data, as well as the inherent stochastic errors in texture analysis and batch-to-batch variations in dough preparation, four widely used kernel functions in food texture modeling were evaluated and compared: the Squared Exponential (SE) kernel, Matern 5/2 kernel, Rational Quadratic (RQ) kernel, and the RQ + White composite kernel. Specifically, the SE kernel serves as a baseline stationary kernel suitable for smooth, continuous data; the Matern 5/2 kernel is adept at modeling mechanical testing data with less stringent smoothness requirements; the RQ kernel is capable of capturing nonlinear relationships across multiple scales; and the RQ + White kernel incorporates a white noise component into the RQ framework to decouple experimental noise from the underlying process-texture regulatory mechanisms.

**Figure 6:**
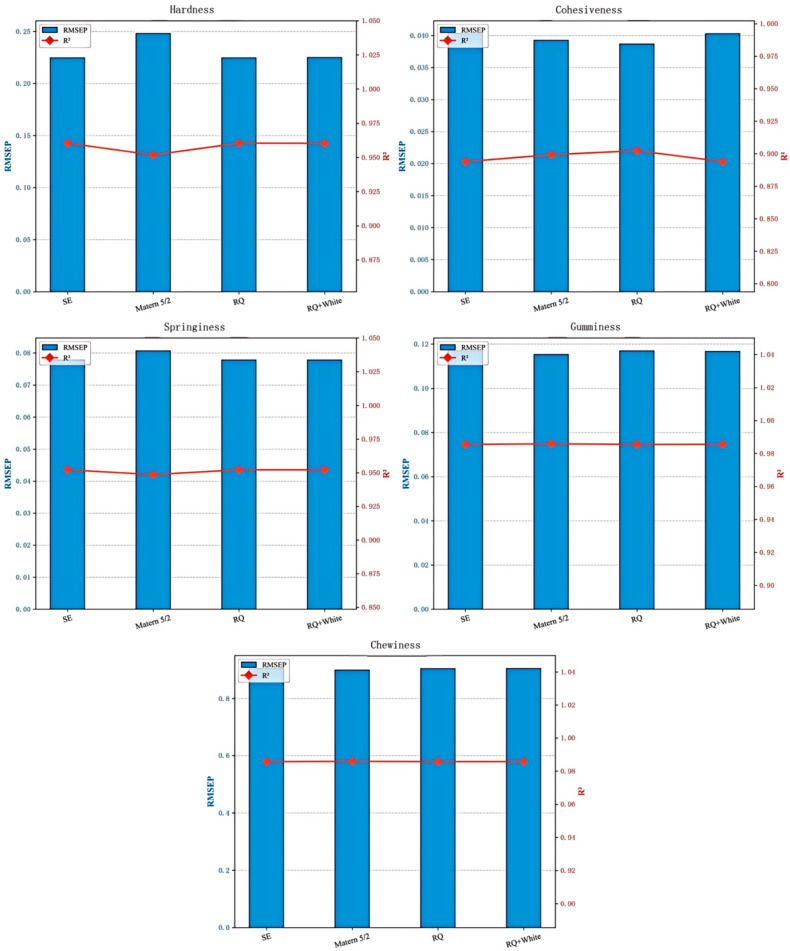
Comparative predictive performance of GPR models utilizing different kernel functions for dough textural properties. Note: Blue bars represent RMSEP (Root Mean Square Error of Prediction, lower values indicate better prediction accuracy), and red lines denote the coefficient of determination (R^2^), values closer to 1 indicate superior predictive performance.

Hyperparameter optimization for the GPR model was targeted at maximizing the Log-Marginal Likelihood (LML). To prevent the optimization from converging to local optima, the optimizer was executed with ten random initializations to ensure the identification of the global optimal hyperparameter configuration. The optimization followed a 4-fold cross-validation strategy identical to that used for the SVR model, with all parameter tuning and model validation conducted strictly within the training set. The independent test set remained entirely isolated from the training and optimization phases to eliminate data leakage and ensure a rigorous, objective comparison between the two models. Following this systematic optimization protocol, the global optimal kernel functions and hyperparameter combinations for the five textural indices were determined. The comparative performance of the four candidate kernels across all indices is illustrated in Figure 6. Performance differences between kernels are statistically stable (from 4-fold cross-validation), confirming the kernel selection is robust for the current dataset.

The results indicate that the optimal kernel functions for various textural indices exhibit a significant dependency on their specific characteristics: basic strength-related indices, such as hardness and springiness, are best modeled by the Squared Exponential (SE) kernel; structural indices sensitive to experimental noise, such as cohesiveness, optimally utilize the Rational Quadratic (RQ) kernel; and composite mechanical attributes, including gumminess and chewiness, achieve peak performance with the Matern 5/2 kernel. Tailoring the kernel function to the specific attributes of each index maximizes the predictive precision of the GPR model. The core hyperparameters established in Table 4, including the optimal kernels and noise variance (α), provide a reproducible framework for subsequent high-precision, single-index textural prediction.

In summary, this section concludes the kernel selection and global hyperparameter optimization for both the SVR and GPR models. By identifying the optimal hyperparameter configurations for each textural attribute, a robust foundation has been established for the subsequent performance comparison and the selection of the improved predictive architecture between SVR and GPR.

### 4.3. Comparative Analysis of Predictive Performance and Fitting Validation for SVR and GPR Models

**Figure 7:**
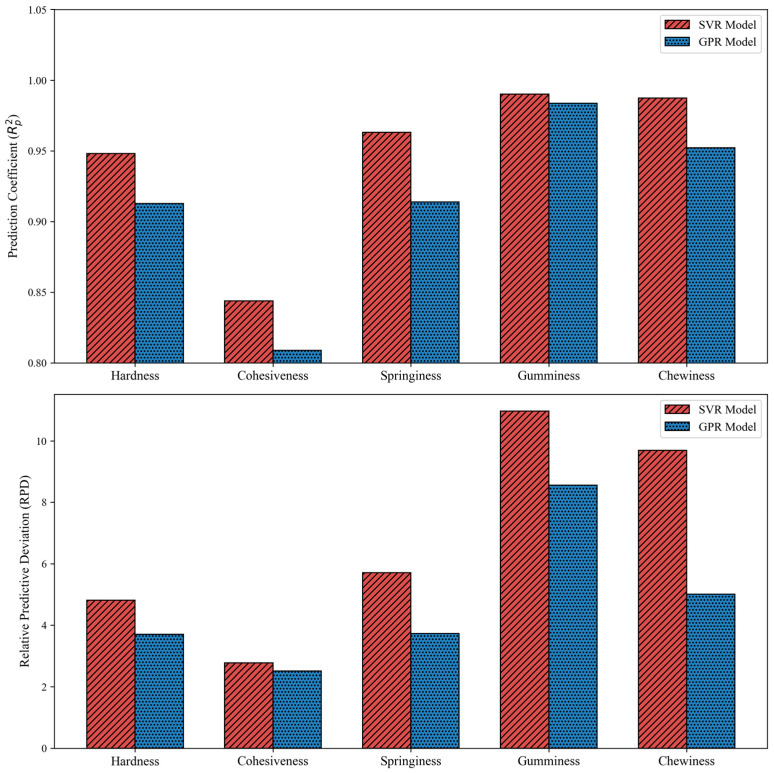
Comparative bar charts of predictive accuracy and robustness between SVR and GPR models.

**Figure 8:**
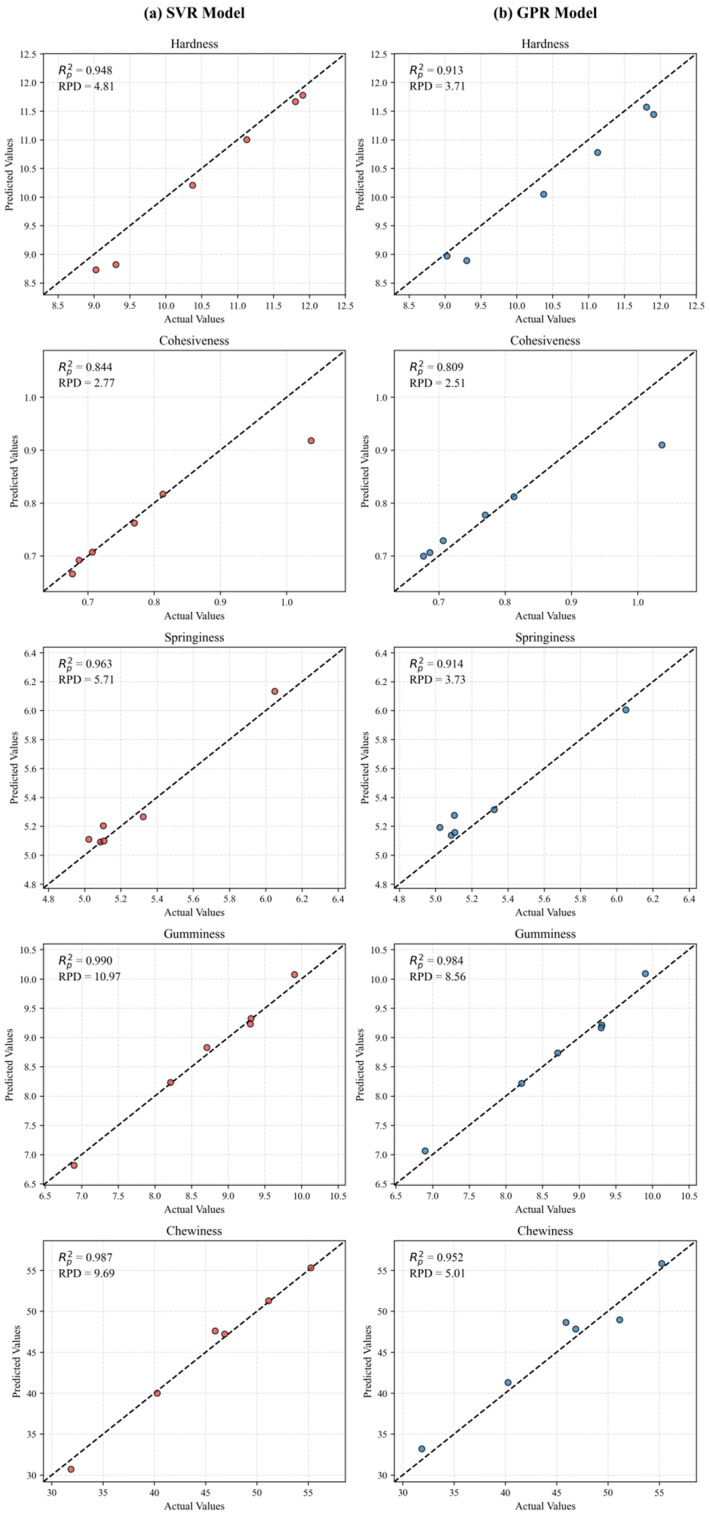
Scatter plots of predicted versus experimental values for the textural properties of 3D-printed dough. Note: The dashed line represents the ideal 1:1 prediction line.

The comprehensive predictive performance and robustness of the SVR and GPR models are quantitatively compared in Figure 7. Both models demonstrated exceptional generalization capabilities on the independent testing set, with $R_{p}^{2}$ values consistently exceeding 0.84 and RPD values reaching up to 10.97. Furthermore, the 1:1 scatter plots (Figure 8) visually confirm that the predicted textural values for both models tightly cluster around the ideal diagonal line. This proximity between predicted and actual values underscores the high precision of the kernel-based algorithms in mapping the complex relationship between KGM concentration, printing pressure, and the resulting dough texture.

As illustrated in Figure 8, the visual fitting performance of the two models is entirely consistent with the quantitative metrics. For the SVR model, all data points are positioned closely along the ideal *y = x* diagonal, exhibiting a tight distribution without significant outliers or systematic bias. Particularly for gumminess, chewiness, and springiness, the predicted values nearly coincide with the experimental observations, providing visual evidence of its favorable fitting precision and robustness. In contrast, the GPR model displays a more dispersed distribution, with certain parameters (e.g., hardness, springiness, and cohesiveness) showing slight deviations from the ideal diagonal. This deviation is most pronounced for cohesiveness, further corroborating that its predictive accuracy is slightly inferior to that of the SVR model. The SVR and GPR models exhibit complementary performance characteristics for texture prediction. The SVR model demonstrates superior interpolative accuracy for deterministic point predictions, while the GPR model provides unique value in quantifying predictive uncertainty.

**Figure 9:**
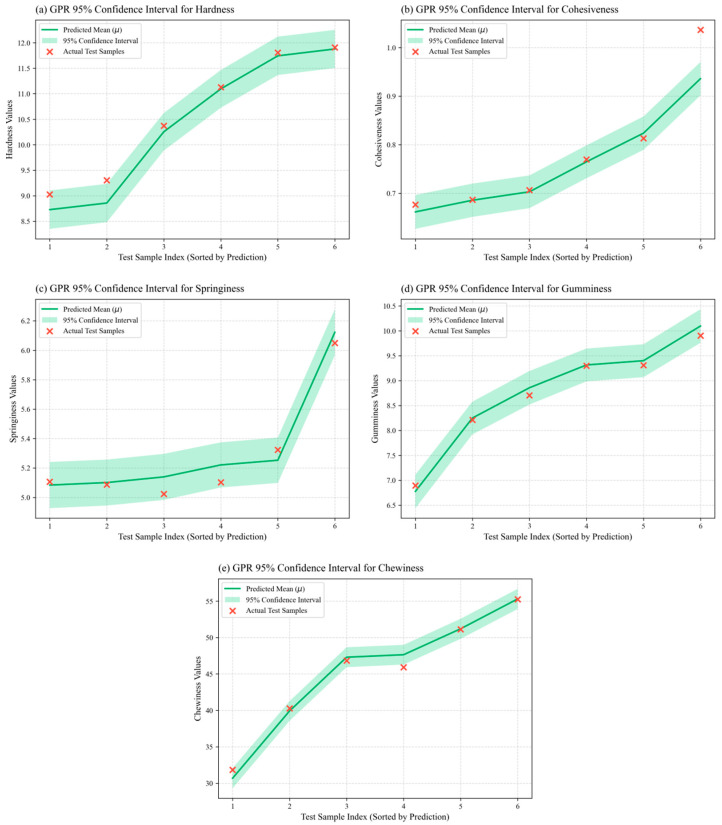
95% confidence intervals and uncertainty analysis for textural predictions using the GPR model.

As illustrated in the uncertainty analysis (Figure 9), out of the total 30 independent test predictions across the five textural parameters, 28 points fell strictly within the 95% predictive intervals, yielding an empirical coverage rate of 93%. This quantitative coverage generally aligns with the theoretical confidence level. It is important to note that an idealized 100% absolute coverage often indicates overly wide, conservative, and uncalibrated uncertainty bounds, particularly when data is limited (n = 30). By strictly calibrating the noise level bounds within the GPR kernel, the optimized model prevents overconfident extrapolations while maintaining realistic constraints. The statistically expected exclusion of a few individual points effectively demonstrates that the model does not blindly expand its bounds; instead, the intervals autonomously widen in sparse formulation regions where data is lacking and narrow near densely sampled coordinates, providing an effective method for quantifying predictive uncertainty for novel dough formulation design. Although the GPR model moderately increased the noise term to ensure CI coverage—thereby slightly sacrificing predictive precision—it achieved a pragmatic balance between accuracy and uncertainty quantification, providing a probabilistic framework for quality risk control in the industrial scale-up of 3D-printed dough.

The performance disparity between the two models originates from their fundamental algorithmic differences. SVR, grounded in structural risk minimization and utilizing the RBF kernel, effectively suppresses overfitting and minimizes predictive errors within small, nonlinear, and noisy food textural datasets, making it ideal for scenarios requiring maximum quantitative precision. GPR, based on Bayesian inference and adaptive multi-kernel selection, offers slightly lower precision but provides valuable confidence intervals, making it better suited for engineering applications where assessing reliability and quantifying process fluctuation risks are paramount. Within the current dataset, the SVR model shows favorable point-prediction accuracy for dough texture, while the GPR model serves as a complementary tool to characterize predictive uncertainty—their functionalities are complementary rather than competitive.

It is noteworthy that the remarkably high prediction coefficients of determination ($R_{p}^{2}$ > 0.98) obtained for gumminess and chewiness necessitate a critical examination of potential overfitting, particularly given the limited sample size (n = 30). Several methodological safeguards, however, mitigate this concern: (1) all hyperparameter tuning was strictly confined to the training set, and the independent test set remained entirely isolated from any preprocessing or optimization procedures, thereby eliminating data leakage; (2) the 4-fold cross-validation yielded minimal variability in the cross-validated $R_{cv}^{2}$, with a standard deviation (SD) ≤ 0.048 across all textural indices, which indicates stable model performance under different data splits; and (3) the ratio of performance to deviation (RPD) exceeded 2.5 for all textural parameters, reaching values of 10.97 and 9.69 for gumminess and chewiness, respectively—levels that substantially surpass the established threshold for excellent predictive capability in food science. Nonetheless, it must be emphasized that these high performance metrics primarily reflect the model’s interpolation accuracy within the densely sampled parameter space, rather than its capacity for extrapolation beyond the investigated ranges of KGM concentration and printing pressure.

Therefore, SVR is suitable for subsequent process optimization and precise textural forecasting. It is suitable for subsequent process optimization and precise textural forecasting of new formulations, while the GPR model serves as a complementary tool providing critical uncertainty support for the predicted outcomes.

It should be noted that the current predictive framework is a localized model established under fixed environmental and processing constraints (e.g., constant moisture content and nozzle geometry). While it offers high precision for the investigated KGM-gluten system, caution should be exercised when extrapolating these results to formulations outside the defined parameter boundaries. Future studies incorporating a broader range of variables will be essential to enhance the model’s universal applicability.

### 4.4. Model-Based Interpretation of Nonlinear Textural Regulatory Patterns

Utilizing the optimal Support Vector Regression (SVR) model identified in Section 4.3, grid predictions were performed within a parameter space defined by KGM concentration (0–1.0%) and printing pressure (4.0–5.0 bar). Corresponding response surface plots and schematic mechanisms were generated to visualize the nonlinear regulatory effects of these process parameters on the textural indices of 3D-printed dough. It should be clarified that the response surface plots presented herein serve exclusively as a visualization of the SVR model’s predictive outputs, intended to illustrate the captured nonlinear mapping rather than employing traditional Response Surface Methodology (RSM) for fitting or optimization. All subsequent analyses are derived from the predictions of the aforementioned machine learning model.

**Figure 10:**
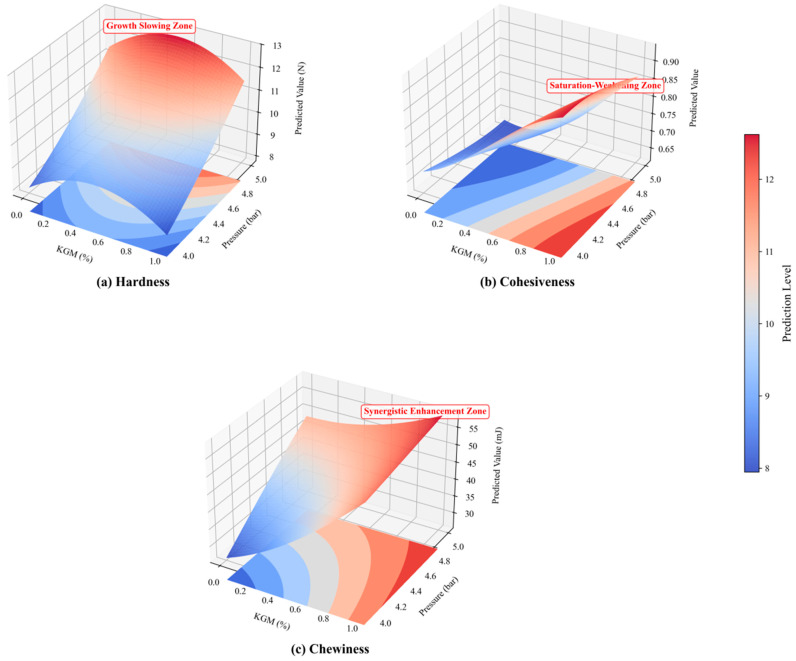
Response surface plots predicted by the SVR model for the textural properties of 3D-printed dough.

The predicted response surfaces (Figure 10) reveal that the impact of KGM concentration on dough cohesiveness and chewiness exhibits characteristic nonlinear patterns. This trend is potentially associated with the complex interactions and moisture distribution variations among KGM [26], gluten proteins, and starch granules, as predicted by the SVR model’s capture of the input-output mapping. The observed nonlinear effect of KGM concentration on dough texture can be explained by established molecular interaction mechanisms that have been validated in previous studies. At lower concentrations, the abundant hydroxyl groups on KGM chains facilitate the formation of physical cross-linking points with gluten proteins via hydrogen bonding [27]. Simultaneously, the high water-holding capacity of KGM enhances the proportion of bound water and restricts moisture mobility, promoting the evolution of the gluten network into a more continuous and dense structure, which significantly enhances cohesiveness, springiness, and chewiness. Beyond the 0.8% threshold, excessive KGM competes with gluten proteins for the limited available water, leading to inadequate gluten hydration. Concurrently, excessive KGM might negatively impact the continuity of the gluten network [28].

The influence of printing pressure on textural indices similarly exhibits pronounced nonlinear characteristics. As shown in the SVR predicted surfaces (Figure 10), hardness, gumminess, and chewiness increased at an accelerating rate within the 4.0–4.6 bar range. Beyond 4.6 bar, however, the growth in hardness decelerated, while springiness exhibited a continuous decline. This trend reflects the underlying “reinforcement-disruption” competitive mechanism induced by extrusion shear within the gluten network. At optimal printing pressures (4.0–4.6 bar), the shear forces within the syringe and nozzle induce the orientational rearrangement of gluten protein chains along the flow direction. Concurrently, the compaction effect increases network density while maintaining structural continuity [29], leading to rapid enhancements in hardness, gumminess, and chewiness, while preserving high springiness levels. When the pressure exceeds 4.6 bar, excessive shear leads to the fracture of gluten protein chains and the cleavage of disulfide bonds, resulting in localized deconstruction and fragmentation of the continuous gluten network. This significantly reduces elastic recovery capacity and causes the growth rate of hardness to plateau due to structural damage. The highly significant negative correlation between springiness and pressure (r = −0.49, *p* < 0.01) reported in Section 4.1, coupled with the predicted deceleration of hardness growth at high pressures in the SVR model, collectively support the interpretation of the “shear-induced disruption” effect from multiple perspectives.

The impacts of KGM concentration and printing pressure on textural properties are not independent but exhibit significant nonlinear coupling effects. SVR predictions indicate that at low KGM concentrations (≤0.3%), the gluten network is inherently weak, making it susceptible to excessive shear damage at high pressures, which limits gains in hardness and springiness. Conversely, within the optimal KGM range (0.5–0.8%), the KGM-reinforced network exhibits enhanced shear resistance, allowing the dough to effectively utilize the compaction effect of higher pressures for textural optimization and demonstrating a “synergistic enhancement” interaction. This interaction is particularly pronounced for chewiness and gumminess [30], suggesting that KGM modulates texture not only directly via water distribution and structural reinforcement but also indirectly by altering the shear responsiveness of the system to printing pressure. These nonlinear coupling features further emphasize that traditional single-factor or orthogonal designs are insufficient to fully capture process-texture relationships, whereas multi-dimensional nonlinear models based on SVR provide a powerful tool for elucidating these complex interaction mechanisms.

In summary, the regulation of 3D-printed dough textural properties by KGM concentration and printing pressure is characterized by distinct nonlinearity. SVR-based analysis further revealed significant nonlinear coupling effects between KGM concentration and printing pressure. We identified a model-predicted favorable processing window of 0.5–0.8% KGM and 4.0–4.6 bar under the current experimental settings. This range provides a quantitative reference for the formulation and process pre-optimization of 3D-printed dough within the investigated parameter space. It should be emphasized that this identified window represents a predictive orientation based on the current experimental data, and its universal applicability across broader formulation boundaries requires further independent validation.

## 5. Conclusions

Addressing the critical requirement for precise textural regulation in 3D-printed dough, this study developed Support Vector Regression (SVR) and Gaussian Process Regression (GPR) models utilizing KGM concentration and printing pressure as core inputs. Following systematic optimization, the SVR model demonstrated robust predictive performance for mapping the nonlinear interactions between formulation and processing parameters, while the GPR model effectively quantified the predictive uncertainty. The model-based analysis revealed significant nonlinear coupling effects between KGM concentration and extrusion shear, and the model predicted a favorable region of 0.5–0.8% KGM and 4.0–4.6 bar within the investigated design space. This predicted window must not be mistaken for a universally optimal formulation. Instead, it represents a data-driven hypothesis that is strictly conditional on the specific experimental settings (fixed moisture content, nozzle diameter, and printing speed). The non-additive interaction revealed—where pressure becomes disruptive only when KGM exceeds 0.8%—is a critical finding, but its quantitative boundaries remain tentative until independently validated with a larger and more densely sampled experimental design. Furthermore, several inherent limitations should be explicitly acknowledged. First, the sample size (*n* = 30) is relatively small, which may restrict the generalizability of the model. The high predictive performance observed is valid only for interpolative predictions within the investigated ranges of KGM concentration (0–1.0%) and printing pressure (4.0–5.0 bar), and extrapolation beyond these parameters is not recommended. Second, the model was developed under fixed moisture content, nozzle diameter, and printing speed; extrapolation to other processing conditions or dough formulations requires further validation and recalibration. Overall, this research offers a preliminary quantitative, data-driven framework for formulation pre-optimization under the current experimental settings; however, as localized predictive models, these findings should be cautiously applied when extrapolating beyond the investigated parameter space.

## Figures and Tables

**Table 1:** Formulations, printing parameters, and experimental groupings of the samples.

Dataset Partitioning	Sample ID	Base Flour Mass (g)	KGM Target Concentration (%)	Actual KGM Mass (g)	3D Printing Pressure (Bar)
Training set	3D-0%KGM	50.0	0.0	0.0000	4.00
		50.0	0.0	0.0000	4.25
		50.0	0.0	0.0000	4.50
		50.0	0.0	0.0000	4.75
		50.0	0.0	0.0000	5.00
	3D-0.25%KGM	50.0	0.25	0.1250	4.00
		50.0	0.25	0.1250	4.25
		50.0	0.25	0.1250	4.75
		50.0	0.25	0.1250	5.00
	3D-0.5%KGM	50.0	0.5	0.2500	4.00
		50.0	0.5	0.2500	4.25
		50.0	0.5	0.2500	4.50
		50.0	0.5	0.2500	5.00
	3D-0.75%KGM	50.0	0.75	0.3750	4.00
		50.0	0.75	0.3750	4.25
		50.0	0.75	0.3750	4.75
		50.0	0.75	0.3750	5.00
	3D-1%KGM	50.0	1.0	0.5000	4.00
		50.0	1.0	0.5000	4.25
		50.0	1.0	0.5000	4.50
		50.0	1.0	0.5000	4.75
		50.0	1.0	0.5000	5.00
Test set	3D-0.15%KGM	50.0	0.15	0.0750	4.10
Test set	3D-0.35%KGM	50.0	0.35	0.1750	4.40
Test set	3D-0.4%KGM	50.0	0.4	0.2000	4.80
Test set	3D-0.6%KGM	50.0	0.6	0.3000	4.60
Test set	3D-0.85%KGM	50.0	0.85	0.4250	4.90
Test set	3D-0.9%KGM	50.0	0.9	0.4500	4.15

Note: Each independent experimental formulation in the table underwent three parallel physical preparations and texture profile analyses. Specifically, the center point (3D-0.5% KGM, 4.5 bar) includes two additional independent replicate tests to estimate the pure experimental error and stabilize the center region of the model.

**Table 2:** Optimal hyperparameters and performance metrics for the textural attributes of 3D-printed dough using the SVR model.

Textural Properties	Optimal Penalty Coefficient C	Optimal Kernel Parameter γ	Optimal Insensitivity Coefficient ε	$R_{c}^{2}$	$R_{p}^{2}$	RMSEP	RPD
Hardness	316.23	0.1	0.032	0.988	0.948	0.257	4.81
Cohesiveness	1	1	0.01	0.973	0.844	0.049	2.77
Springiness	3.16	1	0.001	0.973	0.963	0.068	5.71
Gumminess	1000	0.032	0.001	0.984	0.99	0.097	10.97
Chewiness	316.23	0.1	0.01	0.996	0.987	0.853	9.69

**Table 3:** Four-fold cross-validation performance metrics of the SVR model for the five textural parameters.

Textural Parameter	$R_{CV}^{2}$	$RMSE_{CV}$
Hardness	0.963 ± 0.030	0.205 ± 0.032
Cohesiveness	0.928 ± 0.044	0.027 ± 0.010
Springiness	0.891 ± 0.033	0.110 ± 0.025
Gumminess	0.946 ± 0.048	0.200 ± 0.027
Chewiness	0.989 ± 0.007	0.700 ± 0.198

**Table 4:** Optimal hyperparameters and performance metrics for the textural attributes of 3D-printed dough using the GPR model.

Textural Properties	Optimal Kernel Function	Optimal Noise Variance (α)	Optimization Algorithm	Number of Restarts	$R_{c}^{2}$	$R_{p}^{2}$	RMSEP	RPD
Hardness	SE	0.001	L-BFGS-B	10	0.9896	0.9604	0.2246	5.51
Cohesiveness	RQ	0.001	L-BFGS-B	10	0.9971	0.9023	0.0387	3.51
Springiness	SE	0.01	L-BFGS-B	10	0.9714	0.9522	0.0778	5.01
Gumminess	Matern 5/2	0.001	L-BFGS-B	10	0.9884	0.9859	0.1152	9.24
Chewiness	Matern 5/2	0.001	L-BFGS-B	10	0.9964	0.9858	0.8996	9.19

## Data Availability

The original contributions presented in this study are included in the article. Further inquiries can be directed to the corresponding authors.
